# Long-Term Feeding of Dairy Goats with Broccoli By-Product and Artichoke Silages: Milk Yield, Quality and Composition

**DOI:** 10.3390/ani10091670

**Published:** 2020-09-16

**Authors:** Paula Monllor, Raquel Muelas, Amparo Roca, Alberto S. Atzori, José Ramón Díaz, Esther Sendra, Gema Romero

**Affiliations:** 1 Departamento de Tecnología Agroalimentaria, Escuela Politécnica Superior de Orihuela (EPSO), Universidad Miguel Hernández de Elche, 03312 Orihuela, Spain; pmonllor@umh.es (P.M.); raquel.muelas@umh.es (R.M.); aroca@umh.es (A.R.); jr.diaz@umh.es (J.R.D.); esther.sendra@umh.es (E.S.); 2 Dipartimento di Agraria, Università Degli Studi di Sassari, 07100 Sassari, Italy; asatzori@uniss.it

**Keywords:** circular economy, alternative feedstuffs, milk yield, fatty acid profile

## Abstract

**Simple Summary:**

In the Mediterranean region, artichoke and broccoli are major crops with a high number of by-products that can be used as alternative feedstuffs for ruminants, lowering feed costs and enhancing milk sustainability while reducing the environmental impact of dairy production. However, the nutritional quality of milk needs to be assured under these production conditions. A 40% inclusion of these silage by-products has proven to be a good short-term option (1 month). Therefore, it is interesting to study the effect of feeding animals with these by-products for a longer time, such as full lactation (6 months). With this type of feeding, the performance of the animals fed with the artichoke plant silage was similar to that of those fed with a conventional diet (alfalfa and cereals), even improving the milk quality from the point of view of bioactive compounds. However, the performance of goats fed with broccoli by-product silage in high amounts worsened due to a low intake of the diet containing that by-product. Therefore, it is desirable to reduce its level of inclusion.

**Abstract:**

The aim of this experiment was to study the effects of a 40% inclusion of broccoli by-product (BB) and artichoke plant (AP) silages in dairy goat diets on the milk yield, composition and animal health status during a full lactation. Feed consumption was lower in AP and BB animals due to their composition and higher moisture content, and BB animals showed a significant reduction in body weight. Milk from the BB treatment had the highest fat content, total solids and useful dry matter content (5.02, 13.9 and 8.39%, respectively). The Se level was slightly lower in AP and BB animals; however, the milk of these treatments was the lowest in Na and, in the case of BB animals, the richest in Ca (1267 mg/kg). Control and AP milk showed a similar fatty acid profile, although AP had a more beneficial aptitude for human health (lower ratio of n6/n3, 12.5). Plasma components, as metabolic parameters, were adequate for goats. It was concluded that a 40% inclusion of AP is an adequate solution to reduce the cost of feeding without harming the animals’ health or performance and to improve the nutritional milk quality. It is necessary to lower the BB level of inclusion to increase feed consumption.

## 1. Introduction

According to the Food and Agriculture Organization (FAO) [1], the production of goat milk is in third place (behind cow and buffalo), with a world production of over 18,600,000 t. The main goat milk-producing country worldwide is India, while in Europe the countries that top the list of the highest producers are France, Greece and Spain. Murciano-Granadina is one of the most cosmopolitan Spanish goat breeds [2] and is used on farms in various countries in Europe, Africa and South America. This breed is generally exploited in semiextensive systems under different climatic conditions, feeding on natural pastures or different crop stubble throughout the year, receiving supplementation in critical periods based on agricultural by-products or conventional feeds [3]. Goat’s milk is a rich source of nutrients in the human diet that can influence the prevention of certain types of cancers and cardiovascular diseases due to its Se and polyunsaturated fatty acids (PUFAs) contents, such as vaccenic and rumenic acids or conjugated linoleic acid (CLA) [4,5].

Worldwide artichoke (*Cynara scolymus* L.) and broccoli (Brassica oleracea var. Itálica) productions are important, as they reached values of 1,505,328 and 25,984,758 t of artichoke and broccoli, respectively [1]. These crops generate large amounts of waste that represent an environmental problem due to their rapid decomposition [6]. Wernli and Thames [7] indicate that artichoke plant can generate a yield of 11.1 t/ha of forage, so 1,300,000 t/year of this feedstuff could be available for ruminant feeding. Regarding broccoli, Ros et al. [8] calculated that 29.5% of the total broccoli harvested consists of stems and inflorescences not suitable for human consumption and they could be considered a concentrate rather than a forage, due to their low fibre content and high protein level [9].

The use of by-products for ruminant feeding allows their transformation into high quality food for humans, such as meat and milk [10], while promoting the development of the circular economy and reducing the use of land and water for food production and decreasing competition between animals and humans [11]. However, the marked seasonality and high water content of these feeds limit their systematic use as fresh components in animal feeding.

The silage of these by-products manages to achieve the appropriate fermentation conditions to ensure the nutritional quality and safety necessary to be part of the ration of small ruminants [12,13,14] while allowing their conservation for prolonged periods of time (up to 200 days according to Monllor et al. [14]). The references found in the literature on the effect of silage consumption of these by-products on milk quality and composition, as well as on the health status of the animals, are scarce and tested in the short term [12,15,16,17]. Only three studies have been published about its use in dairy goats. Muelas et al. [18] observed that the inclusion of artichoke plant silage of up to 25% in the diet had no effect on the sensory properties of yogurt made from the milk of these animals, while Monllor et al. and Monllor et al. [19,20] observed that the inclusion of up to 40% of artichoke plant silage and broccoli by-product in the middle stage of lactation (fourth to fifth month) had no relevant effects on the milk yield, composition and technological properties, whereas the inclusion of 60% of the by-product reduced performance.

Broccoli by-product and artichoke plant silages were tested in a previous short-term study for 5 weeks [20], showing favourable results. With the present experiment, we aimed to study during a full lactation the effect of the inclusion of 40% (on a dry matter basis) of the same by-product silages in the ration of dairy goats on the milk yield, composition, mineral and lipid profile and health status of the animals.

## 2. Materials and Methods 

### 2.1. Animals and Facilities

Murciano-Granadina goats from the experimental farm of the Miguel Hernández University were allocated with a straw bed, access to outdoor yards, free access to water and enough feeder space for all animals (at least 35 cm/animal). Animals were fed twice a day, at 8:00 and 14:00, and milked once a day (Casse milking parlour, 2 × 12 × 12, GEA, Germany), as usual in the region. The experiment was carried out between the months of May and October. This study was approved and authorized by the Ethical Committee of Experimentation of the Miguel Hernández University (code UMH.DTA.GRM.01.15), authorized by the competent public body (Conselleria de Presidència i Agricultura, Pesca, Alimentació i Agua of Generalitat Valenciana, Spain).

### 2.2. Experimental Design

From a group of 80 goats at the onset of lactation (4th week) fed with a conventional diet (control, C), a pre-experimental individual sampling was performed and 72 animals were selected, with an average body weight (BW) of 44.6 ± 7.81 kg, an average milk yield of 2.43 ± 0.21 kg/day and a somatic cell count (SCC) of 5.14 ± 0.55 Log cells/mL. The animals were divided into three groups with homogeneous characteristics regarding the commented variables. Each group was assigned to one treatment: control (C), which represents the conventional diet of the region (alfalfa hay and a mixture of grains) and two treatment rations that included 40% (on dry matter basis) of broccoli by-product silage (BB) or artichoke plant silage (AP) in a total mixed ration (TMR). Silages were manufactured with a baler-wrapper (Agronic MR 820, Netherlands), with a weight of 300 kg and without additives, according to the work published by Monllor et al. [14]. All rations were calculated according to the formulation recommendations of Fernández et al. [21]. They were isoenergetic and isoproteic and the daily amount offered was 2.22 kg DM/day. BB and AP silages had a lower cost than other feedstuffs used in animal feeding (45 and 42 EUR/t, respectively). Table 1 and Table 2 show the amounts of the proportions of ingredients in each diet, as well as costs and the composition and mineral and lipid profiles. Feeding was performed in a group to avoid the stress caused by the individual confinement of the animals during a long-term study (6 months), as was done by Nudda et al. [22], due to the gregarious behaviour of goats [23]. This experimental design allows the average food consumption of the animals to be determined, although not the exact amount of individual feed intake nor ingested nutrients. However, the facilities and devices present on the farm allowed the collection of individual milk samples.

**Figure 1:**
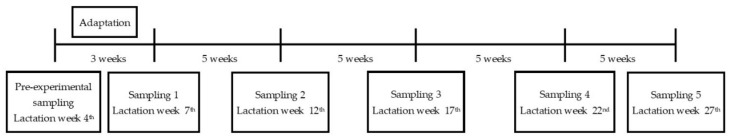
Chronogram of the experiment.

Once the pre-experimental sampling was done, the experiment lasted 23 weeks. The first three weeks for each group served as time to adapt to their diet. In the following 20 weeks, data on BW and milk yield were taken, and blood and individual milk samples were collected for a subsequent laboratory analysis every 5 weeks, with 5 samplings in total during the whole experiment (Figure 1). In addition, the same week of individual sampling, after the milking of each experimental group, milk samples from the refrigerating tank were collected on three consecutive days for an analysis of the mineral and lipid profile. Then, the leftover milk was transferred to another refrigerated tank to get an empty and clean tank to receive milk from the next experimental group.

### 2.3. Variables Analysed

BW (kg) was determined by weighing animals with a precision scale of 100 g (APC, Baxtran, Spain). Feed consumption data were taken from two consecutive days in each sampling week and calculated as the average of the difference between the feed amount offered and refused, determining the dry matter by dehydration in an oven at 105ºC for 48 h of a representative sample of the feed amount refused by the animals for each treatment. Representative samples were taken from each silage and ration at the start of the experiment for a subsequent laboratory analysis. To avoid providing the animals with spoiled silage, each bale was removed after two days of being open. In this way, it was possible to feed the animals with silage in optimal conditions that would not have been affected by aerobic degradation. The composition of the rations was determined in a similar way to Monllor et al. and Monllor et al. [19,20], using Association of Official Agricultural Chemists (AOAC) methods [25] for dry matter (DM, g/kg; method 930.5), organic matter (OM, g/kg DM; method 942.05), ether extract (EE, g/kg DM; method 920.39) and crude protein (CP, g/kg DM; method 984.13). The contents of neutral detergent fibre (NDF, g/kg DM), acid detergent fibre (ADF, g/kg DM) and acid detergent lignin (ADL, g/kg DM) were analysed according to Van Soest et al. [26]. The total polyphenol content (TP, g/kg DM) was determined by the Folin–Ciocalteu method reported in Kim et al. [27]. The proportion of short chain volatile fatty acids (VFAs, g/kg DM)—acetic, propionic and butyric acid, also including lactic acid and ethanol—were determined by high performance liquid chromatography (HPLC) (Agilent 1200 and Supelcogel C-610H column: 30 cm × 7.8 mm ID; [28]). The apparent in vitro dry matter digestibility (IVDMD, g/kg DM) was determined in duplicate by the method of Menke and Steingass [29]. An analysis of the fatty acid profile in the diets was carried out by direct methylation on the lyophilised samples, without prior extraction of the fat, according to Kramer et al. [30]. Methylated fatty acid esters (FAMEs) from diets were identified and quantified by a gas chromatograph (GC-17A Shimadzu, Japan) coupled with a flame ionisation detector (FID) equipped with a capillary column (CP Sil 88 100 m × 0.25 mm internal diameter and 0.20 µm internal coverage, Agilent, USA). A FAME standard mix (18912-1AMP, Sigma-Aldrich, USA) was used to identify the fatty acids present in the samples.

For the analysis of dietary and milk minerals, the same procedures were followed as in Monllor et al. [20], with a previous digestion of the samples performed according to González Arrojo et al. [4]. Na, Mg, K, Ca, P, S (g/kg DM) and Se, Zn, Cu, Fe and Mn (mg/kg DM) were determined by prior microwave digestion (Ethos Easy, Milestone, Italy) and identified with an ICP-MS octupole chromatograph (Agilent 7500 Reaction System, USA), using an internal standard.

The dairy yield and macrocomposition were determined similarly to Monllor et al. [20]. The milk production of each animal (kg/day) was recorded during milking using a Lactocorder^®^ device (Lactocorder, Switzerland), which collected a 100 mL individual sample for subsequent analyses. The milk macrocomposition (fat, protein, useful dry extract, UDM; true protein, casein, whey protein, lactose, total solids, TSs; nonfat total solids, NFTSs; ash; %) and urea content (mg/L) was analysed by medium infrared spectroscopy (MilkoScan™ FT2, Foss, Denmark) calibrated for goat milk. The somatic cell count (SCC, Log cell/mL) was determined by an electronic fluoro-optical method (DCC, DeLaval, Sweden). The fat corrected milk yield was calculated according to the Gravert equation [31]: fat corrected milk (FCM) (3.5%) = 0.433 × yield (kg/day) + 16,218 × fat yield (kg/day), and fat and protein corrected milk yield according to the equation of Schau and Fet [32]: FPCM = yield (kg/day) × (0.337 + 0.116 × fat (%) + 0.060 × protein (%)).

Analysis of the milk fatty acid profile was carried out in duplicate, with an extraction using the Folch method with some variations reported in Romeu-Nadal et al. [33] and a subsequent methylation according to the method of Nudda et al. [34], similar to what was done by Monllor et al. [21]. The chromatograph, the column and the FAME mix for the identification of the milk fatty acids were the same as those used in the diets. The indices related to the nutritional quality of milk fat were calculated—the Atherogenicity Index (AI) and Thrombogenicity Index (TI)—according to Ulbricht and Southgate [35], and the Desaturase Index (DI) for C14:0, C16:0 and C18:0, according to Lock and Garnsworthy [36].

On the same day that the milk sampling was performed, blood samples were taken from fasting animals for an analysis of glucose, urea, β-hydroxybutyrate (BHB), cholesterol, nonesterified fatty acids (NEFAs) and haematocrit, similarly as published by Monllor et al. [20]. Blood samples were analysed by enzymatic spectrophotometry. For glucose and cholesterol (mg/dL), a glucose oxidase/peroxidase kit was used (Refs. 11,503 and 11,505, Biosystems, Spain); for urea (mg/dL), the kinetic method GN 10,125 developed by Gernon was used (Spain); for the BHB (mmol/L), the Ranbut D-3-Hydroxybutyrate kit (RB 1007, Randox, UK) was used, and for the NEFAs (mmol/L), an enzymatic-spectrophotometric method FA 115 (Randox, UK) was used. The haematocrit (%) was determined with a microhaematocrit.

### 2.4. Statistical Analysis

SCC values were transformed into logarithm base 10 to carry out the statistical analysis. A normality test was performed on data (PROC UNIVARIATE. SAS v9.2, 2012) and the result was that all variables complied with normality and variance homogeneity. The variables obtained from individual animals were analysed according to a mixed linear model with repeated measures (PROC GLIMMIX. SAS v9.2, 2012), introducing into the model the covariate of the data obtained in the pre-experimental sampling, according to the following equation:

*Y* = *µ* + *Di* + *Wj* + *DixWj* + *covY0* + *Ak* + *e*, (1)

where *Y* is the dependent variable, *µ* is the intercept, *Di* is the fixed effect of the diet (*i* = C, BB, AP), *Wj* is the fixed effect of the lactating week (*j* = 7, 12, 17, 22, 27), *DixWj* is the interaction of the diet with the lactating week, *covY0* is the effect of the value of *Y* in control 0, Ak is the random effect of the animal and *e* is the residual error. For each variable, the covariance model that presented lower AIC and BIC statistics was used.

In the case of the milk lipid and mineral profile variables, an ANOVA (PROC. GLM, SAS v9.2, 2012) was performed, similar to the previous one, except that the random effect of the animal was not considered.

## 3. Results

No significant differences (*p* > 0.05) between groups were observed in the pre-experimental sampling (fourth week of lactation) for any of the variables analysed (body weight, milk yield and composition, SCC and milk mineral and fatty acid profile).

### 3.1. Body Weight and Milk Performance

**Figure 2:**
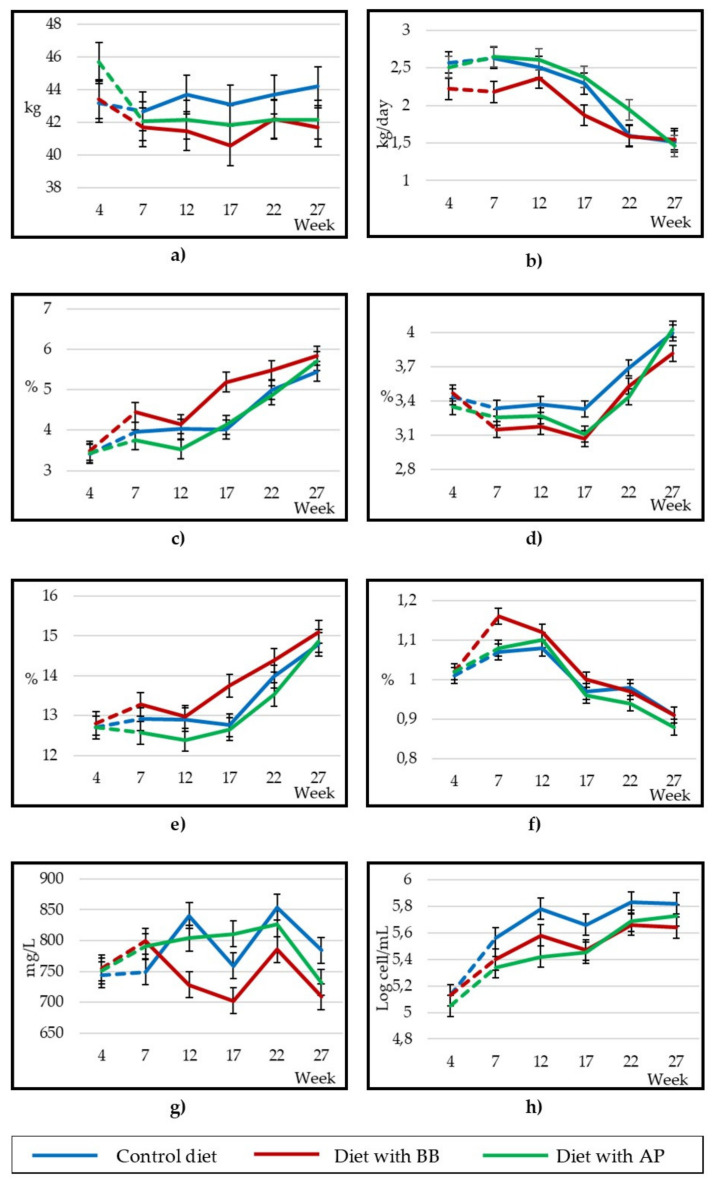
Comparison of changes in body weight (**a**), milk yield (**b**), milk fat (**c**), milk protein (**d**), total solids (**e**), ash (**f**), milk urea (**g**) and somatic cell count (**h**) in goat milk (24 goats per group) from 7th to 27th lactation week.

BW remained constant in animals fed with AP throughout the experiment, and a significant increase (*p* < 0.01) was observed in C animals, while BW of animals fed wit BB fluctuated throughout the experiment, with a decrease in the first half (weeks 7th to 17th) before reaching similar levels at the end of the experiment to those at the outset (Figure 2a). The C group had the highest average BW in the treatment (43.5 kg; Table 3), with significant differences (*p* < 0.01) with the BB group (41.5 kg), while the AP group did not show differences compared to any of the other treatments (42.1 kg). The average feed consumption was higher in the C group (1.98 ± 0.112 kg DM/day), followed by AP animals (1.82 ± 0.151 kg DM/day), while a remarkable lower consumption in BB animals (1.59 ± 0.124 kg DM/day) was observed. All three treatments reduced feed consumption mid-lactation: 160 and 180 g DM/day less between weeks 12th and 17th in C and AP animals, respectively, and 100 g DM/day less between weeks 17 and 22 in BB animals.

The milk yield was slightly lower in the BB group (1.91 kg/day, Table 3) compared to the AP group (2.21 kg/day, *p* < 0.05), with no differences in the C group (2.11 kg/day) and with a significant and gradual decrease (*p* < 0.001) throughout the experiment in the three treatments (Figure 2b), although in a different way, which meant the interaction of the treatment with the sampling was significant. The milk yield of animals fed with BB decreased markedly in the first sampling (week 7), increased in week 12 to levels close to the rest of the treatments and then decreased again significantly at week 17, with the three treatments reaching similar values at the end of lactation (Figure 2b); the AP group’s milk yield decreased gradually throughout the experiment, and some oscillations were observed in the C group at the end of the experiment (week 22). Regarding the FCM and FPCM, no differences were observed between treatments in terms of mean values (Table 3); the value of both parameters decreased in the C and AP treatments as lactation progressed (*p* < 0.001), while in the BB treatment, the FCM remained (data not shown).

Regarding the milk macrocomposition, BB presented a higher average fat content (*p* < 0.01) (0.5% more, in absolute terms) than the other treatments (Table 3). A gradual increase (*p* < 0.001) in the fat content was observed in the three treatments throughout the experiment, generally with an inverse evolution to that observed in the milk yield, and the fat of the BB group was significantly greater than the other treatments at week 17 (Figure 2c), while in the rest of the samplings no significant differences (*p* > 0.05) were observed between treatments. The C treatment presented the highest content of crude and true protein (3.55 and 3.29%, respectively; *p* < 0.05. Table 3), while the BB treatment presented significantly lower values (3.35 and 3.12%) without significant differences between the two treatments with AP (3.42 and 3.18%). As observed with fat, the crude protein content increased (*p* < 0.001) as lactation progressed, and C presented a significantly higher content (*p* < 0.001) than the other treatments in weeks 17 and 22. The milk protein contents of the C and AP animals were equal at the end of the experiment (Figure 2d), but higher (*p* < 0.05) than in BB animals. In relation to whey protein, the C and AP groups were significantly higher than the BB group (0.431 and 0.404 vs. 0.356%, respectively; *p* < 0.001). In relation to TSs and UDM, BB animals presented significantly and slightly higher contents (13.9 and 8.39%, respectively; *p* < 0.05) than AP animals (13.2 and 7.79%), with no differences of both treatments in C animals (13.5 and 8.05%). These variables evolved in a similar way as fat and protein did throughout lactation (Figure 2e). There were no significant differences in the average content of ash between treatments and it decreased (*p* < 0.001) over the weeks (Figure 2f). C and AP animals presented higher (*p* < 0.05) milk urea contents (797 and 793 mg/L, respectively) than BB animals (745 mg/L) and the evolution throughout the experiment was different between treatments—more constant in AP and with 90 mg/L maximum oscillations in C and BB animals, as can be seen in lactation weeks 12 and 17 in Figure 2g. The SCC increased (*p* < 0.001) in the three treatments as lactation progressed (Figure 2h), with a higher content in C animals (5.73 Log cells/Ml; *p* < 0.05) than BB and AP animals (5.55 and 5.53 Log cell/mL, respectively).

### 3.2. Milk Mineral Profile

The only significant differences were observed between treatments regarding the contents of Na, Ca, Mn and Se (Table 4). The C group obtained higher (*p* < 0.001) Na and Se contents (378 mg/kg and 17.2 µg/kg, respectively), although with small differences with the other treatments (19 and 47 mg/kg of Na in BB and AP animals, respectively, and 2.8 and 3 µg/kg of Se in BB and AP animals, respectively). BB animals had the highest Ca level (1267 mg/kg; *p* < 0.001) and AP animals showed the lowest concentration of Mn (31.2 µg/kg; *p* < 0.001), with little difference from the other treatments (Table 4). In relation to the evolution of each mineral throughout the experiment, a reduction in the concentration of Cu was observed (−27.5 µg/kg), the levels of K and Zn fluctuated during lactation without significant differences between the beginning and the end and, in the rest of the minerals, an increase was observed over lactation in the three treatments—68 mg/kg Na, 40 mg/kg Mg, 204 mg/kg P, 72 mg/kg S, 89 mg/kg Ca, 16.1 µg/kg Mn, 69 µg/kg Fe and 4.1 µg/kg Se—as the average increases from the beginning to the end of the experiment.

### 3.3. Milk Fatty Acid Profile

**Figure 3:**
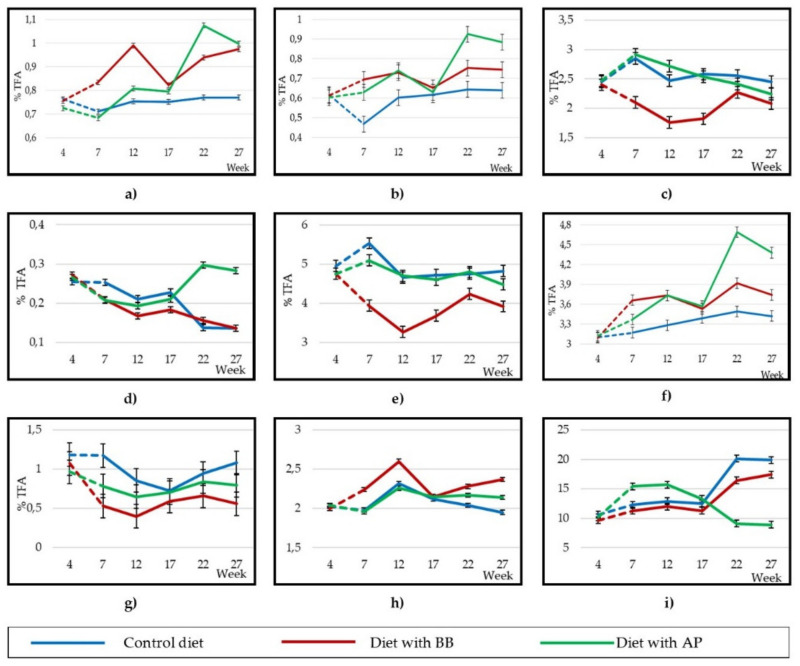
Changes in goat milk lipid profile from refrigerated tank (3 samples/sampling and treatment) during the experiment: C15:0 (**a**), C17:0 (**b**), C18:2*n6* (**c**), C18:3*n3* (**d**), polyunsaturated fatty acids (PUFA) (**e**), odd and branched chain fatty acids (OBCFA) (**f**), ∑CLA(**g**), SFA/unsaturated fatty acid (UFA) (**h**) y *n6/n3* (**i**).

The greatest differences in the fatty acid contents were found in C15:0, C16:1cis9 and C17:0, where BB and AP animals presented the highest concentrations during the experiment (Figure 3a,b), and in some isomers of oleic acid, such as vaccenic (C18:1trans11) and rumenic acids (C18:2cis9,trans11), in which the highest concentration corresponded to the C animals (Table 5), although all differences were of small magnitude. As lactation progressed, the differences between treatments in linoleic acid (C18:2n6) and PUFA contents (Figure 3c,e respectively) were reduced. However, from week 17, α-linolenic acid (C18:3n3) and the ratio n6/n3 (Figure 3d,i) increased and the AP group obtained better results (higher level of α-linolenic acid and lower ratio n6/n3).

Regarding the fatty acid groups, BB animals presented the worst results from the point of view of functional compounds for human health—a lower content of CLAs, higher ratio of saturated fatty acids (SFAs)/unsaturated fatty acids (UFAs) and a higher AI and TI values (Table 6). The C group had the highest level (*p* < 0.001) of the sum of CLA isomers (0.954 g/100 g of total fatty acids (TFA)), followed by the AP group (0.752 g/100 g TFA) and BB group (0.547 g/100 g TFA). The MUFA content was significantly (*p* < 0.0001) higher in C (27.5 g/100 g TFA) than in AP (27.1 g/100 g TFA) and BB (26.2 g/100 g TFA). The PUFA content was similar in C and AP animals (4.89 and 4.73 g/100 g TFA, respectively), and both were significantly (*p* < 0.0001) higher than BB animals (3.81 g/100 g TFA). The SFA/UFA ratio was significantly (*p* < 0.0001) lower in C animals (2.08), followed by AP animals (2.14) and was significantly higher in BB animals (2.33). However, the n6/n3 ratio was lower (*p* < 0.0001) in AP animals (12.5), followed by BB animals (13.7) and higher in C animals (15.6). Related to odd and branched chain fatty acids (OBCFA), the silage treatments showed a higher content than the C group (3.35 g/100 TFA; *p* < 0.001), with higher contents in the AP compared to the BB group (3.95 and 3.71 g/100 TFA, respectively). The effect of lactation and diet x sampling interaction was also significant in OBCFA, because although in C animals it remained constant, in AP animals it increased in week 7 and later in week 17, and in BB animals it increased in week 22 (*p* < 0.001; Figure 3f). Regarding the evolution of the summations throughout the experiment, the three treatments showed similar changes, although at some points of the experiment small oscillations occurred (Figure 3e,g,h), except in the case of the ratio n6/n3 (Figure 3i), which decreased markedly and significantly in the AP group from week 17th, due to the increase in C18:3n3 (Figure 3d).

### 3.4. Plasmatic Metabolites Profile

**Figure 4:**
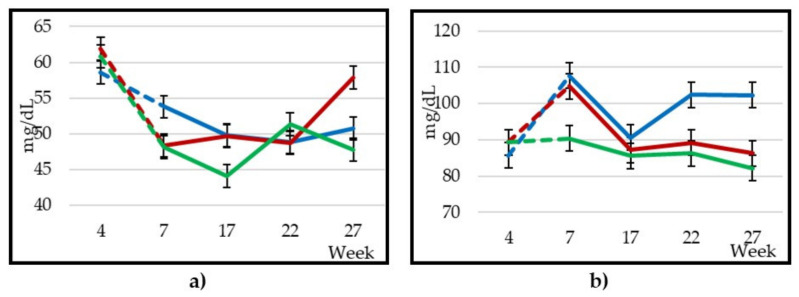

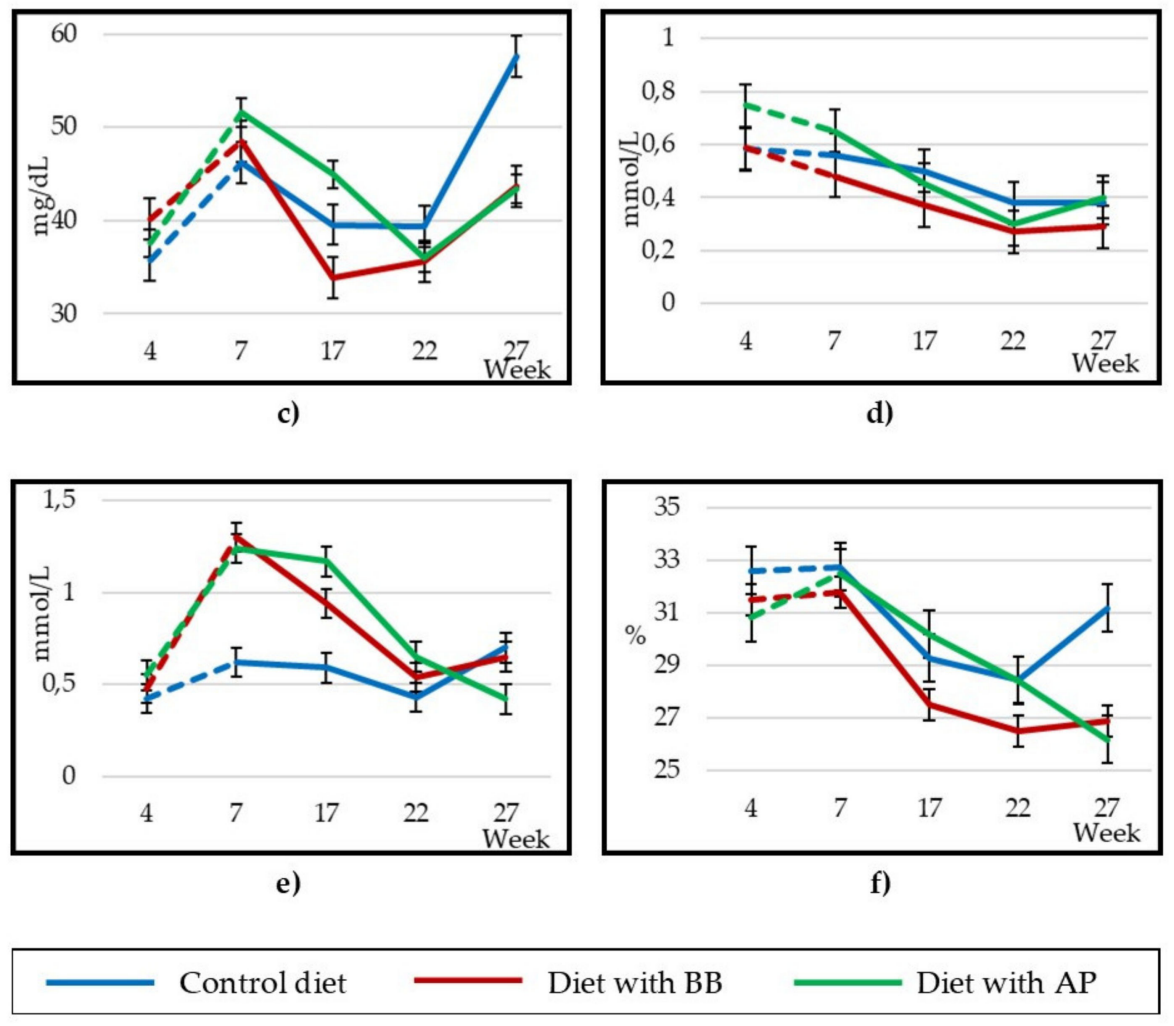
Changes in plasmatic metabolites (24 goats per group) throughout the experiment: glucose (**a**), cholesterol (**b**), urea (**c**), BHB (**d**), NEFA (**e**) and haematocrit (**f**).

Significant differences (*p* < 0.001) were observed between treatments (Table 7 and Figure 4). The glucose content decreased (*p* < 0.001) in C and AP animals during the first part of the experiment (Figure 4a) and stabilised later in C animals, while in BB animals it increased at the last sampling and oscillated in the last two samplings in AP animals (weeks 22 and 27). Regarding cholesterol, the C group reached levels significantly higher (100 mg/dL; *p* < 0.0001) than the BB and AP groups (91.9 and 86.1 mg/dL, respectively). The cholesterol levels of the C and BB groups decreased from lactation week 7 (*p* < 0.001) (−17.09 and −17.55 mg/dL, respectively; Figure 4b). C animals’ cholesterol increased again later, while BB animals remained unchanged until the end; AP animals did not show significant differences between any sampling. The plasma urea content was slightly higher in C and AP animals (45.7 and 44.0 mg/dL, respectively) compared to BB animals (40.4 mg/dL), and the evolution throughout the experiment was similar in the three treatments (Figure 4c), except between weeks 17 and 22, where plasma urea content of animals fed with C and BB remained constant and decreased in AP animals. Finally, the urea content increased in the three treatments at the last sampling, although more markedly in C animals (+18.3, +8.1 and +7.4 mg/dL in C, BB and AP animals, respectively). Diet had a significant effect on the BHB concentration: the average value of the BB group was lower than the C and AP groups (0.356 vs. 0.456 and 0.450 mmol/L, respectively for BB, C and AP groups; *p* < 0.01). BHB levels decreased during the experiment, with slight fluctuations (Figure 4d). The NEFA level was higher in BB and AP animals at the start of the experimental phase (Figure 3e), and an increase in these treatments was observed since by-product silages were included in the diets, as the three treatments had similar levels in the pre-experimental period (0.424, 0.476 and 0.550 mmol/L, respectively for C, BB and AP animals). The NEFA levels of BB and AP animals decreased (*p* < 0.001) gradually throughout the experiment until they reached a similar level to C animals at week 22. Haematocrit decreased in all three treatments over the whole experiment (Figure 4f), and a slight increase was observed at the last sampling in C animals, which caused significant, although minor differences (*p* < 0.01) compared to BB animals, and without differences compared to AP animals (30.4, 28.2 and 29.3% for C, BB and AP animals, respectively).

## 4. Discussion

### 4.1. Body Weight and Milk Performance

The BW reduction observed in the first sampling (week 7 of lactation) was due to the fact that the animals had the greatest lactation needs at the same time, which did not coincide with the time of the maximum intake capacity, which encourages weight loss [37]. BW increased later, coinciding with the reduction in milk yield and the recovery of feed consumption typical of this lactation stage. BB animals showed a lower feed consumption than C and AP animals, because the diet that included BB contained a higher concentration of fermentation products, such as acetic acid, ethanol and ammonia N (Table 1), which had a depressive effect on feed consumption, as observed by Huhtanen et al. [38] through a meta-analysis of 240 studies. As in Monllor et al. [20], the higher moisture content of the BB diet and, therefore, the higher ration volume, decreased consumption. Other causes that affected feed consumption were the higher content of polyphenols in BB, which limits intake, as observed by Oliveira et al. [39] in growing calves and in grazing Sardinian goats, as shown by Decandia et al. [40]. The average feed consumption of C and AP animals (1.98 and 1.82 kg DM/day) was higher than that reported by Criscioni and Fernández [41] in Murciano-Granadina goats (1.7 kg DM/day) with an average milk yield of 2.2 kg/day and a BW of 46 kg, fed with alfalfa and concentrate. BB consumption was slightly lower than the values commented (1.59 kg DM/day).

The milk yield values observed in this experiment are similar, or even slightly higher in the case of the AP group, to those obtained from the equation proposed by León et al. [3] to model the lactation curve for Murciano-Granadina goats: y = 1906 + 0.0229t − 0.000254t^2^ + 0.000264 (t − 57.28)^2^, where y is the daily production (kg/day) and t represents the days in lactation. The inclusion of BB silage at the level tested (40% DM in the diet) negatively affected the milk yield, as a marked decrease was observed in this treatment compared to C and AP groups in the first 17 weeks in lactation, although after the 22nd week, the differences between treatments disappeared. On the other hand, the AP group’s milk yield was not significantly different from C group’s, which indicates that the inclusion of 40% of artichoke plant silage during lactation allows for the maintenance of the productive level.

The higher fat content in BB animals’ milk (5.02%) was due to its lower milk yield, also related to the higher NEFA content observed, so it indicated a greater tendency for a negative energy balance, probably due to a greater mobilisation of body reserves [42]. Even though the level of NEFA in AP animals was similar to that in BB animals (0.868 mmol/L), the higher milk yield (2.21 kg/day) of the AP group meant that fat concentration was higher in the BB group than the AP group. Another cause that explains the higher fat content of BB animals’ milk is the higher concentration of acetic acid (9.50 g/kg DM) in the diet, which served as a precursor for the “de novo” synthesis of fat in the mammary gland [43]. Lough et al. [44], when they supplied a continuous intravenous infusion of acetate in dairy goats at different times of lactation, did not observe changes in milk yield or protein content. The higher fat content of BB animals’ milk explains the higher level of TSs and UDM (13.9 and 8.39%, respectively) also observed in this treatment, even though C and AP animals had higher protein values.

The SCC increased as lactation progressed and the yield reduced, as occurred in Strzałkowska et al.’s study [45]. The SCC values are within normality for Murciano-Granadina goats with a correct health status of mammary gland [46].

### 4.2. Milk Mineral Profile

The mineral profile of this study coincides with that shown by Stergiadis et al. [47]. The Ca/P ratio of BB and AP animals is similar to that observed in local breeds in southern Europe [48,49]. The Ca/P ratio of C animals was lower due to its lower Ca content. The higher concentration of Ca in BB animals is due to the fact that the diet of this treatment also presented a higher level of Ca than the other two [50], which is beneficial for milk coagulation and curd firmness [51].

### 4.3. Milk Fatty Acid Profile

OBCFA levels were within the normal range for milk, between 2 and 3% of total fat, according to Patel et al. [52]. These same authors found a higher concentration of OBCFA in milk from cows fed grass silage, indicating that the more NDF content in the diet, the more OBCFA will be synthesised by ruminal microorganisms due to a higher production of VFAs. This could be the explanation for the higher total OBCFA content in AP animals, as well as C11:0, C17:1 cis9, iso C13:0, iso C14:0, iso C15:0 and iso C17:0. The higher levels of C15:0 and C17:0 in BB animals’ milk come from a greater reserve mobilisation in the animals of this treatment, as these fatty acids are synthesised de novo from adipose tissue [53]. The initial increase (week 7) of OBCFA in AP animals could also be due to a mobilisation of reserves, coinciding with the peak of lactation; however, the OBCFA content stabilised in this group and the next increase that occurred at week 17 may already have been due to an adaptive response to the higher NDF content of the AP diet following the microbial origin of these fatty acids.

The lower concentration of linoleic, vaccenic and rumenic acids in BB was because the diet that included this by-product contained a lower proportion of linoleic and α-linolenic, precursors that are biohydrogenated in the rumen to form the aforementioned acids [54]. The Short Chain Fatty Acid (SCFA) concentrations in this study are similar to those found by Arco-Pérez et al. [55] in milk from Murciano-Granadina goats fed with a tomato by-product silage and to those commonly found in cow’s milk [56]. This means that the inclusion of these by-product silages in the goat diet at the tested concentrations (40%) seems to have little impact on the flavour of milk, due to the role that SCFAs play in the organoleptic properties of milk [57]. From week 17 of lactation, the nutritional quality fatty acid profile of AP improved from the point of view of human health, because of a lower *n6/n3* ratio [58], which gives added value to the milk of goats fed with the silage, due to the current high demand for high quality healthy products for human consumption.

### 4.4. Plasmatic Metabolites Profile

Glucose levels coincided with those observed by Hamzaoui et al. [59] in goats of the same species. The glucose concentration was higher at the beginning of the experiment due to gluconeogenesis that takes place at the beginning of lactation [60], whereby the animals are able to reach the peak of production despite the limited intake capacity that occurs in this stage. The same could be the cause of the increase in glucose in BB animals at the last stage of the experiment, due to the lower feed consumption of the animals in this treatment, which caused the mobilisation of reserves and the reduction in BW. In addition, higher contents of TP in BB animals could cause an improvement in carbohydrate catabolism, as Makkar et al. and Zhong et al. [61,62] observed when glucose levels increased as the content of catechins and condensed tannins were higher in goat diets. The beneficial effects of TP on the lipid metabolism of small ruminants have been also demonstrated [63]. As a result of the lower feed consumption in AP and BB treatments in the stages with the highest energy demands, the NEFA content was higher in these treatments due to the mobilisation of adipose tissue to meet the animals’ requirements [64,65]. These differences in NEFAs were not accompanied by differences in BHB, due to the availability of glucose (a result of the glycogenesis observed in BB), which is used to oxidise NEFA in the liver and produce energy [66]. As the level of feed consumption was adjusted to the milk yield, the concentration of NEFA was reduced, as occurred in Ríos et al. [67].

With the inclusion of BB in the diet, the cholesterol level increased between the 4th and 7th week of lactation, as in the C group, due to the synthesis of lipoproteins responsible for the transport of lipids to the mammary gland, habitual in this lactation stage [68]. This increase in cholesterol at the onset of lactation has been corroborated by other authors, such as Ruas et al. and Guedon et al. [69,70], who established that in beef cattle the minimum cholesterol level occurred at birth and the maximum was reached nine weeks later. The blood cholesterol content decreased as lactation progressed and milk production decreased, according to the Wood model [71]. However, it increased in the C group from week 17, probably due to a positive energy balance (excess of energy intake compared to the level of production at that time), as observed by Ríos et al. [67], coinciding with the increase observed in BW. The cholesterol levels observed in this experiment are within the normal range of values (69.2–239 mg/dL) proposed by Merck et al. [72], in all treatments, indicating that the diets were adequate for the observed production levels.

The plasma urea level was higher at the onset of lactation due to the energy deficit typical of this stage [66]. The increase in urea in C animals at the end of the experiment is due to an excess of protein ingested in the ration [73], which, together with the excess of ingested energy discussed above, would indicate that the energy/protein balance was adequate, but the total amount of feed consumed was higher than the animals’ requirements. This can be confirmed by the fact that the significant increase in the body weight of the goats fed the C diet (also compared to the by-products containing diets) at the end of the experiment. The average urea content of BB animals was lower than that of the other treatments due to the lower protein intake along with the higher TP content of this diet [74].

Taking into account the cost of the AP ration presented in Table 1, the feeding was reduced by 14.64% compared to diet C (76.19 EUR and 89.26 EUR/animal, respectively, during the 23 weeks that the study lasted), without major changes in the milk yield and composition, and with the benefit of a healthier lipid profile for human health, as already explained. On the other hand, in the BB diet, the feeding expenditure was even lower (66.56 EUR/animal), although with the consequent reduction in milk yield.

## 5. Conclusions

The use of artichoke plant silage in diets of dairy goats in the tested proportion (40% on a dry matter basis) over a full lactation did not lead to marked differences in milk yield and quality or compromise the animal health status compared to the diet commonly used in intensive farms (alfalfa hay and mix of cereals and legumes). Regarding the lipid profile, from a nutraceutical point of view, the milk of goats fed with AP, especially at the end of lactation, is more beneficial as it has a lower *n6/n3* ratio. AP is therefore considered an efficient alternative for the use of residues derived from the agro-industrial sector, which allows the production of animal feed of high biological value for humans, with potential benefits for human health.

Regarding the inclusion of broccoli by-product silage in 40% DM in the diet during a full lactation, with a composition similar to that tested in this study, it could generate a dietary selection that is superior to what is desired, which would reduce the feed intake and lead to a lower milk yield, and at the same time promote a lower recovery of body reserves at the end of lactation, together with worse AI and TI indices in milk. Therefore, it is concluded that it is convenient to study the inclusion of a lower amount of this ingredient in the ration, especially during the peak of lactation, or the presentation of BB in other ways that stimulate its intake when it is part of a TMR, in order to maintain animal performance while benefits for the circular economy are achieved.

## Figures and Tables

**Table 1:** Ingredients and chemical composition of the experimental diets provided to goats of the present study.

Item	Diets		
	C	BB	AP
**Ingredients (g/100 g DM)**			
Alfalfa hay	37.4	12.0	-
Oat	16.0	32.0	13.5
Barley	8.00	3.00	6.40
Corn	9.0	3.00	8.00
Dried sugar beet pulp	7.50	3.00	6.50
Sunflower meal	3.40	1.00	3.00
Peas	3.00	1.00	2.30
Cottonseed	3.00	1.00	2.30
Soybean meal 44%	4.50	1.00	11.0
Corn DDGS	3.00	1.00	2.50
Sunflower seeds	2.00	1.00	2.00
Beans	1.40	0.500	1.10
Wheat	1.00	0.300	1.00
Soy hulls	0.500	0.200	0.400
Silage	-	40.0	40.0
Cost (EUR/kg DM)	0.28	0.26	0.26
**Chemical Composition**			
DM (g/kg FM)	893	336	450
g/kg DM			
OM	935	904	901
EE	41.9	39.1	35.2
CP	162	164	164
NDF	376	354	381
ADF	243	227	238
ADL	56,5	48.0	49,3
TP	3,65	4,93	3,28
IVDMD	744	737	668
^1^ ME (Mcal/kg DM)	2.37	2.36	2.29
**VFA and Fermentative Metabolites (g/kg DM)**			
Lactate	0.00	40.8	49.4
Acetate	0.00	9.50	1.39
Ethanol	0.00	9.22	1.58
Ammonia N	0.03	0.410	0.09

C: Control diet; BB: Diet that includes broccoli by-product silage; AP: Diet that includes artichoke plant silage; DM: Dry matter; FM: Fresh matter; OM: Organic matter; CP: Crude protein; CF: Crude fibre; NDF: Neutral detergent fibre; ADF: Acid detergent fibre; ADL: Acid detergent lignin; EE: Ether extract; TPs: Total polyphenols; IVDMD: In vitro dry matter digestibility; ME: Metabolisable energy; VFAs: Volatile fatty acids. ^1^ [24].

**Table 2:** Mineral and fatty acid profile of experimental diets provided to goats of the present study.

Mineral Profile	Diets		
	C	BB	AP
Na (g/kg DM)	1.41	5.65	7.33
Mg (g/kg DM)	2.56	2.61	2.58
K (g/kg DM)	16.2	20.9	14.2
Ca (g/kg DM)	8.66	10.30	10.00
P (g/kg DM)	3.36	3.88	4.18
S (g/kg DM)	3.36	4.42	2.85
Se (mg/kg DM)	0.11	0.20	0.13
Zn (mg/kg DM)	40.6	49.8	38.7
Cu (mg/kg DM)	7.61	5.58	7.44
Fe (mg/kg DM)	175	266	272
Mn (mg/kg DM)	36.4	44.1	40.5
**Fatty acid profile (g/100g total fatty acids)**			
C6:0	0.048	0.121	0.108
C12:0	0.150	0.086	0.100
C14:0	0.349	0.374	0.441
C16:0	15.9	18.1	18.4
C16:1 c9	0.31	0.328	0.341
C18:0	3.17	3.16	3.10
C18:1 c9	24.4	36.1	22.7
C18:1 c11	1.04	2.38	1.04
C18:2n6	46.9	28.9	43.7
C18:3n3	5.03	6.09	5.40
C20:0	0.461	0.717	0.914
C20:1n9	0.310	0.447	0.346
C22:0	0.526	0.804	1.17
C23:0	0.123	0.663	0.139
C24:0	0.383	0.580	0.662
SFA	21.8	25.5	25.6
MUFA	26.1	39.3	24.6
PUFA	52.0	35.2	49.8

C: Control diet; BB: Diet that includes broccoli by-product silage; AP: Diet that includes artichoke plant silage; DM: Dry matter; SFAs: Saturated fatty acids; MUFAs: Monounsaturated fatty acids; PUFAs: Polyunsaturated fatty acids.

**Table 3:** Comparison of body weight, milk yield and composition and somatic cell count (SCC) in goat milk, according to the effects considered from 7th to 27th lactation week.

Variable	n	Diets				Significance		
		C	BB	AP	SEM	Diet	Week	Diet × Week
BW (kg)	24	43.5 a	41.5 b	42.1 ab	0.50	**	**	**
Milk yield (kg/day)	24	2.11 ab	1.91 b	2.21 a	0.098	*	***	***
FCM (kg/day)	24	2.40	2.29	2.47	0.098	n.s.	***	***
FPCM (kg/day)	24	2.21	2.07	2.27	0.087	n.s.	***	***
Fat (%)	24	4.49 b	5.02 a	4.40 b	0.157	**	***	***
UDM (%)	24	8.05 ab	8.39 a	7.79 b	0.199	*	***	**
TS (%)	24	13.5 ab	13.9 a	13.2 b	0.21	*	***	**
NFTS (%)	24	8.82	8.73	8.68	0.071	n.s.	***	***
Protein (%)	24	3.55 a	3.35 b	3.42 ab	0.059	*	***	*
True protein (%)	24	3.29 a	3.12 b	3.18 ab	0.052	*	***	*
Casein (%)	24	2.86	2.76	2.77	0.048	n.s.	***	n.s.
Whey protein (%)	24	0.431 a	0.356 b	0.404 a	0.0119	***	***	**
Lactose (%)	24	4.42	4.47	4.41	0.028	n.s.	***	***
Ash (%)	24	1.00	1.03	0.99	0.018	n.s.	***	*
Milk urea (mg/L)	24	797 a	745 b	793 a	15.5	*	***	***
SCC (Log10 cell/mL)	24	5.73 a	5.55 b	5.53 b	0.062	*	***	**

C: Control diet; BB: Diet that includes broccoli by-product silage; AP: Diet that includes artichoke plant silage; SEM: Standard error of mean; BW: Body weight; FCM: Fat corrected milk (3.5%); FCPM: Fat and protein corrected milk; UDM: Useful dry matter content (fat + protein); TSs: Total solids; NFTSs: Nonfat total solids; SCC: Log10 somatic cell count; abc: Least square means within a column having different superscripts differ significantly. * *p* < 0.05; ** *p* < 0.01; *** *p* < 0.001.

**Table 4:** Comparison of mineral profile from refrigerated goat milk tank according to the effects considered from 7th to 27th lactation week.

Variable	n	Diets				Significance		
		C	BB	AP	SEM	Diet	Week	Diet × Week
Na (mg/kg)	15	378 a	359 b	331 c	5.1	***	***	***
Mg (mg/kg)	15	135	138	132	3.6	n.s	***	***
P (mg/kg)	15	1025	992	988	63.0	n.s.	***	**
S (mg/kg)	15	394	381	380	17.8	n.s.	***	**
K (mg/kg)	15	1601	1557	1627	35.2	n.s.	***	***
Ca (mg/kg)	15	1208 b	1267 a	1200 b	26.1	***	***	*
Mn (µg/kg)	15	46.5 a	43.3 a	31.2 b	2.18	***	***	n.s.
Fe (µg/kg)	15	301 a	301 a	277 b	15.7	n.s.	***	***
Cu (µg/kg)	15	72.4	55.7	63.0	5.0	n.s.	***	**
Se (µg/kg)	15	17.2 a	14.4 b	14.2 b	0.540	***	***	***
Zn (µg/kg)	15	3246	3017	3443	213	n.s.	**	**

C: Control diet; BB: Diet that includes broccoli by-product silage; AP: Diet that includes artichoke plant silage; SEM: Standard error mean; abc: Least square means within a column having different superscripts differ significantly. * *p* < 0.05; ** *p* < 0.01; *** *p* < 0.001.

**Table 5:** Comparison of fatty acid composition (g/100 g total fatty acids) measured in goat milk from refrigerated tank according to the effects considered from 7th to 27th lactation week.

Fatty Acids	n	Diets				Significance		
		C	BB	AP	SEM	Diet	Week	Diet × Week
C4:0	15	1.34 a	1.31 b	1.38 a	0.019	*	***	***
C6:0	15	1.86	1.76	1.88	0.047	n.s.	**	*
C7:0	15	0.028	0.030	0.028	0.002	n.s.	***	***
C8:0	15	2.41 b	2.30 c	2.49 a	0.024	***	***	***
4-methyloctanoic acid	15	0.019	0.017	0.020	0.001	n.s.	*	*
4-ethyloctanoic acid	15	0.015	0.017	0.018	0.002	n.s.	*	n.s.
C9:0	15	0.042 b	0.048 a	0.040 b	0.002	**	**	***
C10:0	15	8.10 b	8.14 ab	8.35 a	0.075	*	*	***
C10:1 c9	15	0.027 b	0.033 a	0.027 ab	0.002	*	***	n.s.
C11:0	15	0.236 c	0.259 b	0.278 a	0.004	***	***	***
C12:0	15	3.74	3.83	3.81	0.033	n.s.	***	***
C12:1 c9	15	0.013	0.015	0.015	0.002	n.s.	n.s.	n.s.
iso C13:0	15	0.014 b	0.015 b	0.020 a	0.001	***	n.s.	n.s.
anteiso C13:0	15	0.040 b	0.041 ab	0.045 a	0.001	*	***	***
iso C14:0	15	0.052 c	0.066 b	0.087 a	0.003	***	***	**
C14:0	15	8.49 b	8.73 a	8.82 a	0.044	***	***	n.s.
iso C15:0	15	0.156 b	0.158 b	0.191 a	0.003	***	***	***
anteiso C15:0	15	0.247 b	0.220 c	0.265 a	0.003	***	**	**
C14:1 c9	15	0.124 c	0.142 b	0.153 a	0.002	***	***	***
C15:0	15	0.751 c	0.912 a	0.872 b	0.006	***	***	***
C15:1	15	0.083 ab	0.075 b	0.090 a	0.003	**	***	***
iso C16:0	15	0.202 b	0.240 a	0.233 a	0.004	***	***	**
C16:0	15	23.9 c	26.5 a	24.8 b	0.10	***	***	***
C16:1 t4	15	0.022 b	0.035 a	0.029 ab	0.004	*	*	***
C16:1 t5	15	0.026	0.036	0.031	0.004	n.s.	***	***
C16:1 t6-7	15	0.061	0.036	0.042	0.010	n.s.	n.s.	n.s.
C16:1 t9	15	0.182 a	0.087 c	0.126 b	0.009	***	*	*
C16:1 t10	15	0.020	0.025	0.025	0.003	n.s.	***	***
C16:1 t11-12	15	0.066	0.058	0.059	0.004	n.s.	**	*
C16:1 c7	15	0.228	0.240	0.238	0.006	n.s.	*	***
C16:1 c9	15	0.497 b	0.666 a	0.667 a	0.015	***	***	n.s.
C16:1 c10	15	0.032 ab	0.026 b	0.034 a	0.003	*	***	**
C16:1 c11	15	0.023 ab	0.020 b	0.028 a	0.002	*	n.s.	*
3,7,11,15-Tetramethyl-16:0	15	0.056	0.025	0.029	0.018	n.s.	n.s.	*
Cyclo C17:0	15	0.050 b	0.081 a	0.077 a	0.003	***	***	**
iso C17:0	15	0.341 b	0.305 c	0.363 a	0.005	***	***	**
anteiso C17:0	15	0.325 b	0.347 a	0.328 b	0.004	***	***	**
C17:0	15	0.594 b	0.714 a	0.762 a	0.020	***	***	***
C17:1 c6-7	15	0.045 a	0.037 b	0.043 a	0.002	*	**	***
C17:1 c8	15	0.019 b	0.035 a	0.017 b	0.004	**	n.s.	n.s.
C17:1 c9	15	0.143 c	0.212 b	0.234 a	0.006	***	***	***
Delta C17:2	15	0.021	0.023	0.024	0.002	n.s.	n.s.	n.s.
isoC18:0	15	0.042	0.044	0.046	0.003	n.s.	n.s.	n.s.
C18:0	15	14.1 a	13.4 b	12.4 c	0.10	***	***	***
C18:1 t4	15	0.035	0.029	0.029	0.002	n.s.	***	**
C18:1 t5	15	0.034 a	0.027 b	0.027 b	0.002	*	***	**
C18:1 t6-8	15	0.417 a	0.286 b	0.296 b	0.008	***	***	***
C18:1 t9	15	0.408 a	0.288 c	0.324 b	0.008	***	***	***
C18:1 t10	15	0.572 a	0.318 c	0.411 b	0.013	***	***	***
C18:1 t11 (vaccenic)	15	2.13 a	0.83 c	1.18 b	0.050	***	***	***
C18:1 t12	15	0.550 a	0.388 c	0.427 b	0.011	***	***	***
C18:1 t13-14	15	0.963	0.973	0.553	0.165	n.s.	***	***
C18:1 c9	15	18.9 b	19.5 b	20.2 a	0.21	***	***	***
C18:1 c11	15	0.451 b	0.570 a	0.555 ab	0.038	*	**	**
C18:1 c12	15	0.518 a	0.429 c	0.474 b	0.015	***	***	**
C18:1 c13	15	0.120	0.101	0.102	0.007	n.s.	n.s.	n.s.
C18:1 c14	15	0.493 a	0.415 b	0.388 c	0.009	***	***	***
C18:1 c15	15	0.246 a	0.227 b	0.220 b	0.004	***	***	***
C18:1 c16	15	0.023 a	0.017 b	0.019 ab	0.002	*	*	**
C18:2 t8,c13	15	0.138	0.133	0.139	0.002	n.s.	***	**
C18:2 t9,c12	15	0.027	0.029	0.032	0.003	n.s.	***	**
C18:2 t9,12	15	0.021	0.018	0.021	0.002	n.s.	n.s.	**
C18:2 t10,14	15	0.070 a	0.033 b	0.039 b	0.004	***	n.s.	*
C18:2 t11,c15	15	0.048	0.036	0.046	0.004	n.s.	***	*
C18:2 t11,15	15	0.012	0.008	0.010	0.003	n.s.	n.s.	n.s.
C18:2 t12,c15	15	0.030	0.033	0.030	0.003	n.s.	n.s.	n.s.
C18:2 c9,t12	15	0.121 a	0.109 b	0.117 a	0.002	***	*	n.s.
C18:2 c9,t13	15	0.321 a	0.299 c	0.311 b	0.003	***	***	***
C18:2 c12,15	15	0.026	0.026	0.024	0.002	n.s.	**	**
C18:2n6	15	2.58 a	2.01 b	2.57 a	0.047	***	***	***
C18:2 t9,c11	15	0.048	0.042	0.057	0.003	***	n.s.	*
C18:2 c9,t11 (rumenic)	15	0.843 a	0.443 c	0.619 b	0.018	***	***	***
C18:2 t10,c12	15	0.029	0.030	0.036	0.004	n.s.	n.s.	n.s.
C18:2 t12,14	15	0.020	0.022	0.021	0.003	n.s.	**	n.s.
C18:3n3	15	0.193 b	0.170 c	0.238 a	0.004	***	***	**
C18:3n6	15	0.029 b	0.024 b	0.036 a	0.002	***	n.s.	***
C20:0	15	0.241 c	0.270 b	0.314 a	0.004	***	**	***
C20:1 c5	15	0.021	0.017	0.019	0.002	n.s.	n.s.	n.s.
C20:1 c9	15	0.015 b	0.016 b	0.021 a	0.002	*	*	n.s.
C20:1 c11	15	0.052 a	0.040 b	0.048 a	0.002	***	***	**
C20:1 c15	15	0.016	0.017	0.020	0.003	n.s.	n.s.	n.s.
C20:2n6	15	0.038	0.037	0.038	0.003	n.s.	**	n.s.
C20:3n6	15	0.021	0.016	0.016	0.003	n.s.	*	**
C20:3n9	15	0.079 b	0.075 b	0.104 a	0.004	***	n.s.	**
C20:4n6	15	0.142 b	0.139 b	0.166 a	0.004	***	*	*
C22:0	15	0.033	0.031	0.035	0.003	n.s.	n.s.	n.s.
C22:2n6	15	0.021 b	0.042 a	0.023 b	0.005	**	*	n.s.
C23:0	15	0.023 b	0.034 ab	0.039 a	0.005	*	*	n.s.
C24:0	15	0.031 b	0.043 ab	0.052 a	0.005	**	***	**

C: Control diet; BB: Diet that includes broccoli by-product silage; AP: Diet that includes artichoke plant silage; SEM: Standard error mean; abc: Least square means within a column having different superscripts differ significantly. * *p* < 0.05; ** *p* < 0.01; *** *p* < 0.001.

**Table 6:** Comparison of grouped fatty acids (g/100 g total fatty acids), indices related to cardiovascular health and desaturation activity in goat milk from refrigerated tank according to the effects considered from 7th to 27th lactation week.

Variable	n	Diets				Significance		
		C	BB	AP	SEM	Diet	Week	Diet × Week
SFA	15	67.4 c	69.7 a	67.9 b	0.12	***	***	***
MUFA	15	27.5 a	26.2 c	27.1 b	0.14	***	***	***
PUFA	15	4.89 a	3.81 b	4.73 a	0.067	***	***	***
UFA	15	32.4 a	30.1 c	31.8 b	0.12	***	***	***
OBCFA	15	3.35 c	3.71 b	3.95 a	0.038	***	***	***
∑CLA	15	0.954 a	0.547 c	0.752 b	0.019	***	***	***
SFA/UFA	15	2.08 c	2.33 a	2.14 b	0.013	***	***	***
SCFA	15	13.9 b	13.7 b	14.3 a	0.13	**	***	*
MCFA	15	40.8 c	44.2 a	42.8 b	0.13	***	***	n.s.
LCFA	15	45.3 a	42.1 c	42.9 b	0.20	***	***	*
n3	15	0.193 b	0.170 c	0.238 a	0.004	***	***	**
n6	15	2.83 a	2.27 b	2.84 a	0.047	***	***	***

C: Control diet; BB: Diet that includes broccoli by-product silage; AP: Diet that includes artichoke plant silage; SEM: Standard Error of Mean; SFAs: Saturated Fatty acids; MUFAs: Monounsaturated Fatty Acids; PUFAs: Polyunsaturated Fatty Acids; UFAs: Unsaturated Fatty Acids (MUFA + PUFA); OBCFA: Odd and Branched Chain Fatty Acids; CLA: Conjugated Linoleic Acid; SCFAs: Short Chain Fatty Acids (C6:0 a C10:0); MCFAs: Medium Chain Fatty Acids (C11:0 a C17:0); LCFAs: Long Chain Fatty Acids (C18:0 a C24:0); AI: Atherogenic Index; TI: Thrombogenic Index; DI: Desaturation Index; abc: Least square means within a column having different superscripts differ significantly. * *p* < 0.05; ** *p* < 0.01; *** *p* < 0.001.

**Table 7:** Comparison of plasmatic profile according to the effects considered from 7th to 27th lactation week.

Variable	n	Diets				Significance		
		C	BB	AP	SEM	Diet	Week	Diet × Week
Glucose (mg/dL)	24	50.8 a	51.2 a	47.6 b	0.66	***	***	***
Cholesterol (mg/dL)	24	100.8 a	91.9 b	86.1 b	2.94	***	***	***
Urea (mg/dL)	24	45.7 a	40.4 b	44.0 a	1.14	**	***	***
BHB (mmol/L)	24	0.456 a	0.356 b	0.450 a	0.028	*	***	***
NEFA (mmol/L)	24	0.585 b	0.856 a	0.868 a	0.048	***	***	***
Haematocrit (%)	24	30.4 a	28.2 b	29.3 ab	0.47	**	***	***

C: Control diet; BB: Diet that includes broccoli by-product silage; AP: Diet that includes artichoke plant silage; SEM: Standard Error of Mean; BHB: β-Hydroxybutyrate; NEFAs: Nonesterified Fatty Acids; abc: Least square means within a column having different superscripts differ significantly. * *p* < 0.05; ** *p* < 0.01; *** *p* < 0.001.
